# Enhancing Working Memory and Reducing Anxiety in University Students: A Neurofeedback Approach

**DOI:** 10.3390/brainsci14060578

**Published:** 2024-06-05

**Authors:** Pierluigi Diotaiuti, Giuseppe Valente, Stefano Corrado, Beatrice Tosti, Chiara Carissimo, Tommaso Di Libero, Gianni Cerro, Angelo Rodio, Stefania Mancone

**Affiliations:** 1 Department of Human Sciences, Society and Health, University of Cassino and Southern Lazio, 03043 Cassino, Italy; giuseppe.valente@unicas.it (G.V.); stefano.corrado@unicas.it (S.C.); beatrice.tosti@unicas.it (B.T.); tommaso.dilibero@unicas.it (T.D.L.); a.rodio@unicas.it (A.R.); s.mancone@unicas.it (S.M.); 2 Department of Medicine and Health Sciences, University of Molise, 86100 Campobasso, Italy; chiara.carissimo@unimol.it (C.C.); gianni.cerro@unimol.it (G.C.)

**Keywords:** neurofeedback training (NFT), working memory enhancement, anxiety reduction, university students, alpha amplitude, cognitive functions, EEG-biofeedback, Emotiv Epoc X, State-Trait Anxiety Inventory (STAI), cognitive performance

## Abstract

(1) Background: Neurofeedback training (NFT) has emerged as a promising approach for enhancing cognitive functions and reducing anxiety, yet its specific impact on university student populations requires further investigation. This study aims to examine the effects of NFT on working memory improvement and anxiety reduction within this demographic. (2) Methods: A total of forty healthy university student volunteers were randomized into two groups: an experimental group that received NFT and a control group. The NFT protocol was administered using a 14-channel Emotiv Epoc X headset (EMOTIV, Inc., San Francisco, CA 94102, USA) and BrainViz software version Brain Visualizer 1.1 (EMOTIV, Inc., San Francisco, CA 94102, USA), focusing on the alpha frequency band to target improvements in working memory and reductions in anxiety. Assessment tools, including the Corsi Block and Memory Span tests for working memory and the State-Trait Anxiety Inventory-2 (STAI-2) for anxiety, were applied pre- and post-intervention. (3) Results: The findings indicated an increase in alpha wave amplitude in the experimental group from the second day of NFT, with statistically significant differences observed on days 2 (*p* < 0.05) and 8 (*p* < 0.01). Contrary to expectations based on the previous literature, the study did not observe a concurrent positive impact on working memory. Nonetheless, a significant reduction in state anxiety levels was recorded in the experimental group (*p* < 0.001), corroborating NFT’s potential for anxiety management. (4) Conclusions: While these results suggest some potential of the technique in enhancing neural efficiency, the variability across different days highlights the need for further investigation to fully ascertain its effectiveness. The study confirms the beneficial impact of NFT on reducing state anxiety among university students, underscoring its value in psychological and cognitive performance enhancement. Despite the lack of observed improvements in working memory, these results highlight the need for continued exploration of NFT applications across different populations and settings, emphasizing its potential utility in educational and therapeutic contexts.

## 1. Introduction

Neurofeedback (or EEG biofeedback) is a non-invasive psychophysiological technique based on the principle of operant conditioning [1,2]. This technique uses changes in brain electrical activity to help individuals regulate the activity or power of specific EEG frequency bands through real-time access to information about their brain electrical activity [3,4]. Thus, it represents true neuro-cognitive training whereby the individual learns to control, regulate, and voluntarily modify their brain electrical activity and to correct EEG alterations and the pathological states associated with them, thanks to immediate feedback provided in a visual/graphic and/or auditory form [5,6]. Generally, brain activity can be measured using various signals that can serve as feedback, such as blood flow, oxygen consumption, and electrical activity. The latter, through the use of EEG, constitutes the most common and utilized form of neurofeedback [7,8,9].

EEG is recorded from the scalp surface, and when pyramidal neurons are activated, it captures the ionic currents in the cerebral cortex due to synaptic excitation of these neurons’ dendrites. The EEG trace results from the alternation between excitatory and inhibitory postsynaptic potentials at these synapses and shows spontaneous activities varying in frequency. Traditionally, these frequencies are divided into five bands, each with its own range, corresponding to different brain states. These bands include delta (1–4 Hz), predominantly observed in deep sleep and in very serious brain disorders; theta (4–8 Hz), commonly associated with drowsiness, childhood, adolescence, and meditation; alpha (8–12 Hz), linked to wakeful relaxation, closed eyes, and the inhibition of cortical activity; beta (12–30 Hz), associated with active thinking, problem solving, active concentration, and anxiety; and gamma (30 Hz and above), related to high-level cognitive functioning and information processing, as well as conscious perception [10,11].

The goal of neurofeedback is thus to teach individuals to regulate their brain activity within a certain frequency band to enhance the associated behavior or mental state [12]. Historically, since its inception in the 1960s, neurofeedback has been used in various fields and for different purposes: for example, as an alternative to pharmacological treatment in individuals suffering from headaches, nausea, and epileptic seizures and as an aid in children with ADHD, who exhibited an unbalanced pattern of brain waves [13,14,15,16,17,18,19].

Due to its positive effects in clinical practice, there has been growing interest in research to verify whether neurofeedback training (NFT) could also positively influence the cognitive abilities of healthy individuals [20]. Some studies seem to support this hypothesis [21]. According to Klimesch (1999), the individual upper alpha band is of great importance for cognitive performance [22].

Based on various studies, researchers have examined the connection between individual alpha wave amplitude (IUA), anxiety, and cognitive performance [23,24]. In these studies, different performance aspects of short-term memory and working memory were assessed with specific tests like the digit span, the N-back and Oddball task [25], and the mental rotation task [26]. Mental flexibility and executive functions were investigated through the Trail Making test [27], and anxiety state through questionnaires like the STAI (State-Trait Anxiety Inventory) [28]. In all these studies, encouraging results emerged on the effectiveness of neurofeedback for the improvement of cognitive and emotional functions. They underscore the intricate relationship between working memory (WM) and anxiety, revealing that anxiety can significantly impair WM performance. Working memory, the cognitive system responsible for the temporary storage and manipulation of information, is crucial for various complex cognitive tasks, including learning, reasoning, and comprehension. Anxiety, particularly in academic settings, tends to disrupt the efficiency of this system, leading to diminished cognitive performance and academic achievement [29].

Neurofeedback training (NFT) has shown the potential to mitigate these effects by enhancing alpha wave activity, which is associated with a state of wakeful relaxation and reduced cognitive interference. For anxiety, the modulation of beta waves through NFT can help reduce symptoms by calming the sympathetic nervous system and reducing the overall arousal levels that characterize anxiety states. By increasing alpha amplitude, NFT may help inhibit the overactivation of neural circuits that are detrimental to working memory due to anxiety. These previous studies have indicated that neurofeedback training (NFT) targeting alpha wave modulation offers significant benefits in managing anxiety and enhancing cognitive functions, particularly working memory. These findings are pertinent to our objectives, as they underline the potential mechanisms through which NFT exerts its effects. Neurofeedback facilitates the self-regulation of brain activity, allowing individuals to consciously influence brain waves associated with cognitive alertness and emotional calmness. For instance, increasing alpha wave activity through NFT has been linked to improved relaxation and reduced arousal levels, which are crucial in high-anxiety states commonly experienced by university students.

The enhancement of alpha activity has been shown to correlate with better performance in working memory tasks. This is attributed to alpha waves’ role in inhibiting distracting stimuli, thus enhancing cognitive focus and information retention. These capabilities are particularly valuable in academic settings where students are required to process and retain large amounts of information under pressure. Therefore, the application of NFT in this context not only supports emotional regulation but also directly contributes to cognitive enhancements that can improve academic performance [30,31,32,33,34,35].

In light of established research demonstrating the efficacy of neurofeedback training (NFT) in modifying alpha activity and reducing anxiety across both clinical and healthy populations, the current investigation extends these findings to a novel context: healthy university students. Despite the prevalence of academic and social pressures experienced by this demographic, which significantly contribute to elevated levels of anxiety and cognitive load, little research has specifically examined the potential cognitive and emotional benefits of NFT within this group. University students are at a critical developmental juncture where cognitive capabilities such as working memory are heavily utilized and are integral to academic success. By focusing on this population, we aim to elucidate the role of alpha activity modulation in cognitive and emotional regulation, potentially offering a non-pharmacological intervention to enhance student well-being and academic performance.

Recent studies have highlighted the significance of alpha waves (8–12 Hz) in cognitive and emotional regulation. Alpha waves are predominantly associated with states of wakeful relaxation and cognitive inhibition, which help minimize distractions and enhance cognitive focus. The choice of electrodes in this study, specifically the parietal (P7 and P8) and occipital (O1 and O2) regions, is based on their established roles in visual processing and attentional control. These regions are crucial for tasks involving working memory, which requires the integration and manipulation of visual and spatial information. By targeting these areas, the NFT aims to increase alpha wave amplitude, promoting an optimal state of relaxation that can enhance cognitive processing and reduce anxiety. The rationale is that elevated alpha activity can inhibit the overactivation of neural circuits involved in stress responses, thereby improving emotional regulation and cognitive performance. This study seeks to extend the understanding of these mechanisms within a population of university students who often experience high levels of cognitive load and anxiety.

In addition to neurofeedback training (NFT), relaxation techniques were incorporated into the intervention to enhance the overall effectiveness of the training. Relaxation techniques, such as guided imagery and mental exercises, have been shown to promote alpha wave activity by inducing a state of calm and reducing cognitive arousal. The synergistic use of NFT and relaxation techniques is hypothesized to facilitate a more robust increase in alpha wave amplitude, thereby optimizing the conditions for cognitive enhancement and anxiety reduction. By combining these methods, the study aims to leverage the immediate calming effects of relaxation techniques with the longer-term neural training provided by NFT, potentially leading to more significant improvements in both working memory and anxiety levels.

### Objectives and Hypotheses

General Objective: To explore the effects of neurofeedback training (NFT) on cognitive and emotional functions in university students. Specific Objective 1: To determine if NFT can improve working memory performance. Specific Objective 2: To evaluate if NFT can reduce levels of state and trait anxiety.

**Hypothesis 1.**  *Neurofeedback training (NFT) combined with relaxation techniques targeting the alpha frequency band will significantly enhance working memory in university students. This hypothesis is grounded in previous research indicating that increased alpha wave activity, facilitated by both NFT and relaxation techniques, is associated with improved cognitive functions, particularly working memory.*

Rationale: Alpha waves (8–12 Hz) are linked to wakeful relaxation and cortical inhibition, which can enhance cognitive processes by reducing interference from irrelevant stimuli. The selected electrodes (P7, P8, O1, and O2) cover parietal and occipital regions associated with visual processing and attentional control, both of which are crucial for working memory tasks. The inclusion of relaxation techniques aims to induce a state of calm that further promotes alpha wave activity.

**Hypothesis 2.**  *NFT combined with relaxation techniques will lead to a significant reduction in state and trait anxiety levels among participants. This reduction is expected due to the combined ability of NFT and relaxation techniques to modulate brain activity in frequency bands associated with relaxation and stress reduction.*

Rationale: Increased alpha activity has been correlated with reduced anxiety and enhanced relaxation. By focusing on the parietal and occipital regions, the NFT aims to elevate alpha waves, promoting a state of calm that can mitigate anxiety symptoms. The relaxation techniques are intended to provide immediate reductions in cognitive arousal, complementing the long-term effects of NFT. The expected direction of the effect is an increase in alpha wave amplitude, which should correspond with decreased anxiety levels.

These hypotheses will be tested using a controlled experimental design, where the experimental group will receive NFT with real-time feedback aimed at enhancing alpha waves in specific brain regions, while the control group will not receive this targeted feedback. The effectiveness of the intervention will be measured through standardized tests for working memory and anxiety before and after the NFT sessions.

## 2. Materials and Methods

### 2.1. Sample

To verify the hypotheses of this study, a statistical power analysis was performed to estimate the sample size using G*Power 3.1 software. Considering a repeated measures ANOVA design with within–between interactions taking into account Group (experimental and control) and Time (six sessions), the following parameters were set: the effect size (ES) was set at 0.30, considered medium according to Cohen’s criteria, an assumed correlation among repeated measures of 0.5, and a significance level α of 0.05. The nonsphericity correction was set to 1, assuming complete sphericity of the measures. The analysis revealed a non-centrality parameter of 24.00, a critical F value of 2.069, with numerator degrees of freedom of 7 and denominator degrees of freedom of 154. The total sample size calculated to achieve adequate power (0.80) was 28 participants. However, the analysis indicated that with this sample size, the actual power of the test reaches 0.960, suggesting a very high probability of detecting a significant effect if present.

Recruitment was carried out by forwarding a request for voluntary participation to the students of the University of Cassino and Southern Lazio, using the informational channels of the internal internship office, through which it is possible to participate in scientific research. The recruitment aimed at ensuring a diverse and representative sample of the student population. Inclusion criteria were as follows: aged between 19 and 26 years; full-time enrollment at the university; and willingness to participate in all sessions and comply with study requirements. Exclusion criteria were as follows: history of neurological or psychiatric disorders; ongoing pharmacological treatment that might affect neurological functioning; and vision impairments not correctable with standard eyewear. An initial screening interview was conducted to verify eligibility, gather basic demographic information, assess understanding of and commitment to the study protocol, and address any potential medical or psychological issues that might contraindicate participation.

A total of 40 students, aged between 19 and 26 years and balanced by sex, thus exceeding the minimum number indicated by the calculation performed with G*Power, participated in the study. After passing the preliminary assessments, candidates provided informed consent before participating. They were informed of their right to withdraw from the study at any time without any consequence. All data were collected with strict adherence to privacy standards and were used exclusively for scientific purposes.

### 2.2. Tools

**Figure 1:**
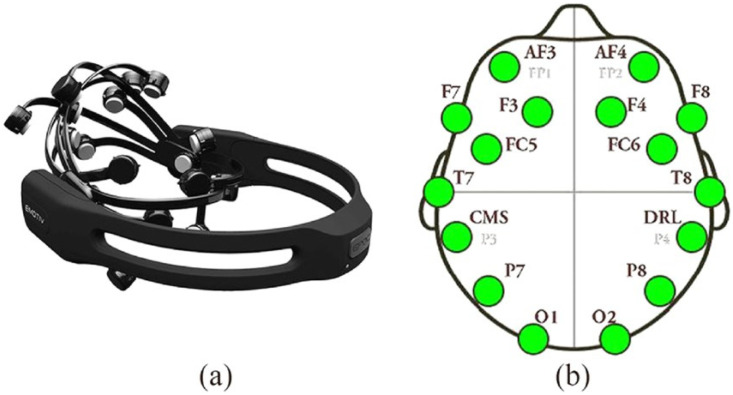
(**a**) Emotiv Epoc X headset; (**b**) Positioning of the 14 EEG channels on the scalp used for neurofeedback training, highlighting the regions of interest for alpha wave enhancement.

**Figure 2:**
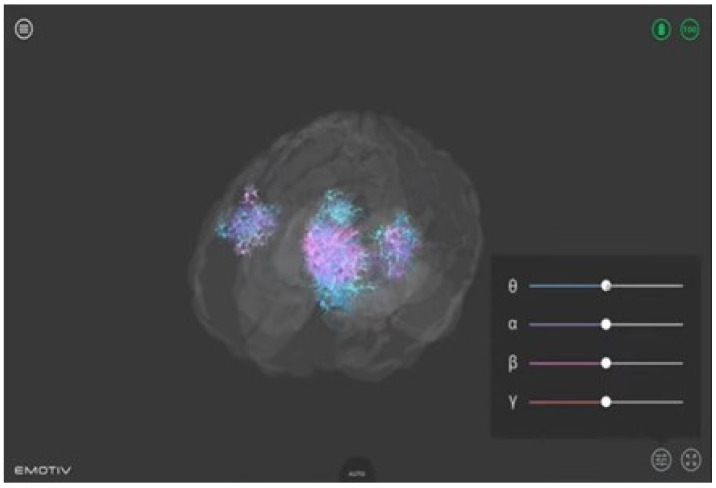
Real-time 3D visualization of brain activity. Color-coded frequency bands captured by the Epoc X device providing participants with visual feedback during neurofeedback training.

For the NFT interventions, the following were used:

- Emotiv Epoc X device (https://www.emotiv.com/epoc/, accessed on 4 March 2024) [36]. This tool is non-invasive as it does not emit any harmful signals to health but is able to detect the EEG signal on the scalp surface using passive saline sensors. The device is easy to use and has not shown any harmful health effects, as supported by recent studies, showing suitable quality in recording the electroencephalographic signal [37,38,39,40]. The device complies with the requirements of the Low Voltage Directive 2006/95/EC, the EMC Directive 2004/108/EC, and the R&TTE Directive 1999/5/EC and has the CE and C-Tick conformity marks [41]. The *Emotiv Epoc X* collects the EEG signal from 14 different channels (AF3, AF4, F7, F3, F4, F8, FC5, FC6, T7, T8, P7, P8, O1, and O2) on the scalp surface, as illustrated by Figure 1. The device is wireless and transmits data via Bluetooth over a 2.4 GHz band with a sampling frequency of 128 bits per second and a bandwidth ranging from 0 to 64 Hz. The associated *Emotiv Pro Lab* software (version 3.0) allows for the processing and encoding of the outgoing EEG signal.

- The Emotiv BrainViz software (version Brain Visualizer 1.1). This software was used to provide visual feedback to the participants. It offers a real-time 3D visualization of the electrical activity recorded by the Epoc X device, as shown in Figure 2. In the visualization, the 4 frequency bands (theta, alpha, beta, and gamma) are color coded, making it possible to visualize their location on the scalp surface in real time. This visualization thus allows one to see both spatial properties (the areas where brain activity occurs) and temporal properties (the type of activity) at the same time [42].

The Emotiv BrainViz software plays a crucial role in our neurofeedback protocol by transforming raw EEG data into a comprehensible and actionable format. This transformation begins with the recording of electrical activity via non-invasive EEG sensors positioned on the scalp. The recorded signals, which primarily capture cortical electrical patterns, are then processed to identify the alpha frequency band, known for its importance in cognitive relaxation and attentiveness. Once the alpha activity is isolated, the Emotiv BrainViz software employs advanced algorithms to render these data into a real-time 3D visualization of brain activity. This visualization is not merely a surface projection but an interpretation of cortical and subcortical regions based on the distribution and amplitude of alpha waves. To enhance the spatial resolution and accuracy of this representation, the software utilizes a technique akin to source localization. This technique involves estimating the origins of brain activity within the cortical structure by using mathematical models that approximate the volume conduction of neural signals through various brain tissues.

The 3D visualization thus provided offers both real-time feedback to the participant and valuable insights for researchers, allowing for immediate adjustments in training protocols to optimize neurofeedback efficacy. By leveraging this sophisticated visual representation, participants can effectively target specific brain areas for alpha wave enhancement, directly linking their cognitive efforts with visual outcomes.

**Figure 3:**
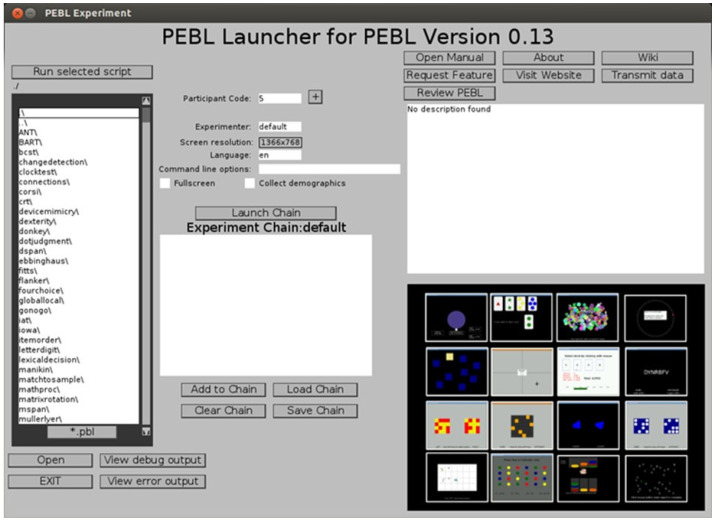
PEBL test platform interface. User interface of the PEBL software platform (version 2.1) used for administering cognitive and memory assessments.

For cognitive tests, assessments were carried out via computerized administration through the PEBL platform [43]. The reference platform is a collection site for various psychometric tests designed to be easily used across multiple operating systems [44]. The current version of the platform is designed to work with PEBL version 0.13 and was released in 2012. It is distributed with PEBL 0.13 and is automatically installed in Documents\pebl.0.13\battery on Windows. Figure 3 shows the test launch screen available on the platform.

The tests used for the assessment were as follows:

Two different working memory tests were employed to comprehensively evaluate the cognitive effects of neurofeedback training (NFT) on participants: the *Memory Span* and *Corsi Block tests*, each serving a specific evaluative purpose tailored to the study’s objectives. The Corsi Block test, administered before and after the entire NFT training sequence—specifically on the first and eighth days—was used to measure broader changes in visuo-spatial working memory across the entire intervention period. This assessment strategy was chosen to capture any enduring cognitive enhancements or adaptations resulting from the neurofeedback sessions, providing insights into the long-term effects of NFT on working memory capacity. Nine blue squares appear on the computer screen, lighting up in a progressively numerical quantity, and the participant must reproduce the exact sequence in which they lit up by clicking on each square with the mouse. It starts with a sequence of 2 squares and continues until the subject can no longer accurately remember the last observed sequence. In contrast, the Memory Span test was administered at both the beginning and end of each of the eight training sessions. This frequent assessment schedule was designed to monitor the immediate, session-specific effects of neurofeedback on verbal working memory. By evaluating participants’ memory performance more regularly, we could track the short-term fluctuations and potential immediate improvements in memory capacity that may occur from session to session. This approach allows for a detailed analysis of the dynamic changes in working memory over the course of the training, offering a nuanced understanding of how each session might contribute to cognitive performance. Memory Span is a simple test to assess short-term memory consisting of 9 stimuli (words and images) presented in rotation. The number of stimuli presented to the subject increases progressively, and the task is to remember as many as possible. It starts with a list of 3 stimuli, and each time the subject correctly recalls all the stimuli in a list, the list increases by one until the subject can no longer remember all the stimuli of the last presented list.

To assess anxiety, the following questionnaire was used:

The State-Trait Anxiety Inventory-2 (STAI-2) [45] is a self-assessment questionnaire where the participant rates, on a 4-point Likert scale (where 1 = not at all and 4 = very much), how much different statements reflect their behavior. This scale consists of a total of 40 questions, 20 for state anxiety (Y1) and 20 for trait anxiety (Y2). The STAI can be administered to individuals or groups and takes 8 min to complete one scale and about 15 min to complete both, allowing for two distinct scores to be derived depending on the aspect being investigated. STAI scores are commonly classified as “no or low anxiety” (scores 20–37), “moderate anxiety” (scores 38–44), and “high anxiety” (scores 45–80). The decision to use STAI over other measures, such as the State-Trait Inventory for Cognitive and Somatic Anxiety (STICSA), was motivated by several factors: (1) The STAI has long been validated and is widely used in both clinical and research settings, providing robust measures of both state and trait anxiety. Its reliability and validity across diverse populations make it a trusted tool for assessing the psychological condition of university students. (2) While STICSA offers a detailed breakdown of the cognitive and somatic aspects of anxiety, our study was primarily focused on the broader psychological impacts of neurofeedback on anxiety. The STAI, which assesses the general feeling of anxiety and its stable occurrence as a trait, was deemed more appropriate for gauging the overall psychological and emotional state changes induced by neurofeedback training. (3) The use of STAI allows for comparative analysis with a broader range of previous studies in neurofeedback and anxiety, many of which have also employed STAI. This compatibility is crucial for situating our findings within the existing research landscape and for potential meta-analyses in future studies.

### 2.3. Procedure

The 40 participants in the pilot study were preliminarily randomized into 2 groups (experimental and control) of 20 individuals each.

The 20 participants in the experimental group were individually administered a 15 min NFT, divided into 5 sessions of 3 min each, interspersed with 1 min of recovery, for 8 consecutive days. In the training sessions, participants in the experimental group were provided precise instructions aimed at enhancing their alpha wave activity, a key focus of our study due to its correlation with reduced anxiety and improved cognitive function. They were asked to enhance alpha waves in the parietal areas P7 and P8 and occipital areas O1 and O2. Specifically, participants were instructed to increase the prominence of the purple color in the 3D brain visualization provided by the Emotiv Brain Viz software, which represented alpha wave activity. To achieve this, participants were guided through a series of mental exercises designed to promote relaxation and mental calmness, thereby elevating alpha wave production. These exercises included focusing on positive memories, engaging in mental imagery of peaceful scenes, and other personalized relaxation techniques that participants found effective. They were continuously monitored and received real-time feedback, allowing them to see the effects of their mental strategies on alpha wave activity.

The aim was to make the purple color, indicative of alpha activity, expand across the 3D visualization of their brain, providing a clear and engaging target for the participants. This interactive and visual approach was intended not only to facilitate the understanding and control of brain activity through neurofeedback but also to reinforce the learning of self-regulation techniques that participants could apply outside of the experimental setting. Thanks to the visual feedback of alpha wave activation, set to the frequency of 7.5–12.5 Hz by the Emotiv BrainViz software (version Brain Visualizer 1.1) [46], subjects could observe the progressive trend of their alpha-type brain activity.

During the neurofeedback sessions, participants in the experimental group received real-time feedback based on their alpha wave activity, visualized through the Emotiv Brain Viz software. This feedback was not limited to passive observation; instead, participants were actively engaged in manipulating their brain activity. The feedback mechanism was designed to be intuitive: the purple color representing alpha activity would intensify and expand across the 3D brain model as alpha levels increased, providing a direct visual representation of brain activity changes.

Participants were instructed that the goal was not necessarily to continuously increase alpha activity throughout the entire session but rather to maintain it within an optimal range that supports relaxation and focused attention. This approach helps in avoiding fatigue and allows participants to stabilize their brain activity at a beneficial level. Training sessions included periods where maintaining a consistent alpha level was emphasized over increasing it, providing a balanced challenge that facilitates learning and adaptation to neurofeedback techniques.

Immediately following each session, participants were asked to describe the mental strategies they employed to control or influence their brain activity. This process was conducted through open-ended questions during a brief, structured interview. The responses were analyzed using qualitative content analysis to identify common themes and strategies across participants. This approach allowed us to understand the types of cognitive processes participants found most effective in manipulating their EEG signals.

The Corsi test and the STAI-2 for assessing memory and anxiety were administered before and after the NFT training on the first and eighth day of the experiment, while the Memory Span test was administered at the beginning and end of each session for the duration of the intervention, i.e., for all eight sessions. Employing both memory tests in these specific ways enabled a comprehensive analysis of working memory modifications due to NFT, capturing both the immediate effects of each session and the cumulative impact over the entire training period. This methodology also provides a robust framework for assessing how different aspects of working memory (visuo-spatial versus verbal) are affected by such cognitive training.

The other 20 participants in the control group were not exposed to visual feedback but only underwent a 15 min EEG recording, again divided into 5 sessions of 3 min each, interspersed with 1 min of recovery. In this case, the procedure was focused only on viewing a graphical representation of a relaxing image depicting a sunny beach with a calm sea projected on the PC screen placed in front of the participants. In this group, the Corsi test and the STAI-2 for assessing memory and anxiety were administered before and after the 15 min EEG recording on the first and eighth day, while the Memory Span test was administered at the beginning and end of each session for the duration of the program, i.e., for all eight sessions.

### 2.4. Description of the General Procedure Action Sequence

Participants were individually summoned (by appointment) to the dedicated experimentation space at the University of Cassino and Southern Lazio in the Folcara Campus. They were welcomed by a first operator in a quiet space, free of noise and distractions, so as to not compromise the validity of the study. After collecting the informed consent of the participants, explaining the experimental procedure to them, and allowing them to become familiar with the environment, the pre-evaluation phase commenced.

Participants were asked to sit on a comfortable chair in front of a computer to perform, with the support of a second operator, the pre-specified computerized pre-tests for the study (assessment of working memory and anxiety).

After completing the pre-evaluation procedure, participants in the experimental groups were accommodated at a second station, placed in front of a PC (whose brightness was set within safety parameters for sight), and a second operator explained the operation of the Emotiv Epoc X device and prepared its placement on the participant’s head, ensuring that the head was not wet, that the subject did not wear metallic objects, and that no excessive movements were made that could interfere with the detection of the electroencephalographic signal, and that the sensors were adequately moistened with saline solution to ensure signal conductivity.

The participant was then provided instructions on how to perform the neurofeedback training with the related EEG recordings.

Once the NFT recording phase (lasting 20 min) was completed, one of the researchers removed the device from the participant’s head and disinfected the sensors and the device for measurement with the next participant.

The same procedure was carried out for participants in the control group, with the difference being that they were not subjected to neurofeedback training but were instructed to observe a relaxing image on the screen (also for a duration of 20 min).

Subsequently, one of the operators had the participant sit back at the starting station to undergo (where appropriate) the cognitive and anxiety tests again. At the end of the session, feedback on the session was requested from the participant, and any observations on the experience were collected. The participant was then dismissed and reminded of the next appointment.

### 2.5. Statistical Analyses

In order to clean EEG artifacts, compute the Fourier transform, and obtain a first matrix of each individual recording, the proprietary Emotiv software named Analyzer (version 3.0) was used. The MATLAB^TM^ computing software (version 23.2) was used to separate the baseline from the recording trace and for the construction of computation matrices for averages in reference to the relative alpha normalized to the reference sample. All other analyses were performed with the IBM Statistical Package for Social Sciences (SPSS version 26) for multivariate and regression analyses.

To evaluate whether the NFT program had significant effects on the variables of the alpha wave, a variance analysis with a mixed factorial model was conducted, which included the time variable (eight sessions) as a factor within participants and the group variable as a factor between subjects. Therefore, for each dependent variable considered, corresponding two-way mixed ANOVAs (8 × 2) were performed. For state and trait anxiety, average scores of State-Trait Anxiety Inventory (STAI-2) before and after the intervention were calculated and analyzed using repeated measures ANOVA. The visuo-spatial working memory test (Corsi Block test) was administered pre- and post-intervention. Consequently, to assess the impact of the NFT, we performed a repeated measures ANOVA focusing on the group (experimental vs. control) and time (pre- vs. post-intervention) as factors. As the Memory Span test was administered at the beginning and end of each of the eight NFT sessions, data were analyzed using repeated measures ANOVA, with time as a within-subject factor across the eight sessions, to monitor changes in memory performance throughout the intervention. Following Cohen [47], partial Eta squared was the measure used to assess the effect size (0.01 = small, 0.06 = medium, 0.13 = large). The significance level was set at *p* < 0.05, while for checking the effects of multiple univariate interactions, a Bonferroni adjustment was introduced by dividing the declared level of statistical significance by the number of dependent variables: *p* < 0.025 (i.e., *p* < 0.05 ÷ 2).

The recorded EEG data were pre-processed using the Emotiv Analyzer and MATLAB^TM^ software to perform automatic artifact removal, separation of the EEG signal into various bands, and calculation of relative alpha for both the experimental and control groups. In this study, relative alpha refers to the proportion of alpha wave activity within the overall EEG signal, a commonly used measure in neurofeedback protocols to assess the effectiveness of training aimed at enhancing cognitive functions and reducing anxiety. The relative alpha values were calculated from the pre-processed EEG by dividing the average amplitude of the alpha band by the average amplitude of the entire EEG band. The relative alpha is calculated using the following formula: Power of Alpha Band/Total Power Across All Bands. This calculation is facilitated by the Emotiv Pro Lab software, which processes the raw EEG data recorded via the Emotiv Epoc X headset. The software isolates the alpha frequency band, typically ranging from 8 to 12 Hz, from the EEG signal and computes its power. The total power is calculated by summing the power across all frequency bands captured by the EEG. The relative alpha metric provides a normalized measure of alpha activity, offering insights into the participant’s state of relaxation and cognitive alertness during and after the neurofeedback sessions. The choice to use relative alpha as a measure is based on its recognized sensitivity to changes in cognitive states induced by neurofeedback, making it a valuable indicator of the training’s effectiveness. By quantifying how much of the brain’s electrical activity falls within the alpha band relative to the entire spectrum of brain waves, we can more accurately assess the impact of our neurofeedback protocol on enhancing cognitive function and reducing anxiety in participants. For the purposes of this study, relative alpha values were specifically obtained from the four recording channels used in the neurofeedback protocol: P7, P8, O1, and O2. These channels were chosen due to their relevance in capturing alpha activity associated with visual processing and attention, areas directly implicated in the cognitive functions targeted by our neurofeedback training. By focusing on these channels, we aimed to precisely measure changes in alpha activity that are most relevant to the desired outcomes of enhanced working memory and reduced anxiety. This selective approach allows for a more accurate assessment of how neurofeedback influences specific areas critical to the regulation of anxiety and cognitive performance rather than a generalized EEG activity across the entire scalp. Regarding relative alpha data, the analysis of studentized residuals demonstrated normality in distribution, assessed by the Shapiro–Wilk test, and the absence of outliers, evaluated by studentized residuals not exceeding ±3 standard deviations. The sphericity of the interaction term was confirmed, as assessed by Mauchly’s test of sphericity (*p* > 0.05).

## 3. Results

### 3.1. Evaluation of the Intervention Effect on Alpha Wave Amplitude

Considering the alpha wave amplitude variable, no statistically significant interaction between Group and Time emerged, F(7,133) = 1.02, *p* > 0.05, partial η^2^ = 0.051. Therefore, simple main effects were evaluated. Significance was detected for the Group variable alone: F(1,19) = 6.05, *p* < 0.05 partial η^2^ = 0.242.

Table 1 below shows the recorded alpha values (indicated in mean, standard error, and confidence interval) obtained in the two groups over the eight days comprising the training. Data shown are averages obtained specifically from the neurofeedback protocol channels P7, P8, O1, and O2.

Subsequently, the pairwise comparisons for each of the eight days were then performed, comparing alpha wave values between the control group and the experimental group. Below, in Table 2, are reported the resulting averages and the *p*-value for each day.

From the results, only day 2 and day 8 showed statistically significant differences (*p* < 0.05) between the two groups in alpha wave amplitude. This indicates that on days 2 and 8, the experimental group exhibited a significant increase in alpha wave amplitude compared to the control group. The other days did not show statistically significant differences.

**Figure 4:**
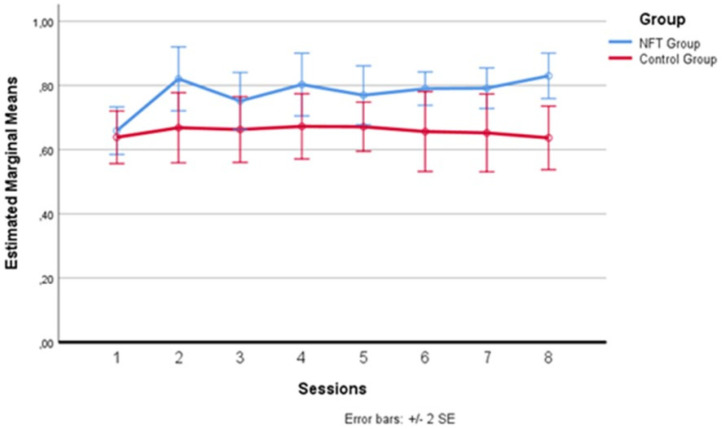
Alpha wave amplitude variation across sessions.

In Figure 4 below, one can more clearly observe the variation in alpha wave amplitude recorded in the two groups during the eight sessions. The average of the intervals comprising each daily training session was calculated for each session. After the baseline recording on the first day, which saw the two groups starting from a substantially homogeneous level, differences emerged from the second day, where the experimental group recorded higher average alpha amplitude levels than the control group. This difference remained evident for all seven days following the first session, with a greater amplitude detectable on the second, fourth, and eighth days, although these specific peaks did not constitute a statistically significant difference between the sessions, configuring a substantial “plateau effect” of the training starting from the second day. This effect has already been documented in the literature [35,48,49], indicating that the increase in alpha does not continue incrementally but tends to maintain a stable level after the start of treatment.

### 3.2. Evaluation of the Intervention Effects on Short-Term Memory

To evaluate the effect of the treatment on short-term memory, measures from the Corsi Block test and the Memory Span test in the two groups were compared. For Memory Span, the repeated measures ANOVA showed significance for the time variable but not for the group: F(1,19) = 85.80, *p* < 0.001, partial η^2^ = 0.819; Table 3 below shows the means, standard error, and confidence interval recorded in the two groups over the eight days.

**Figure 5:**
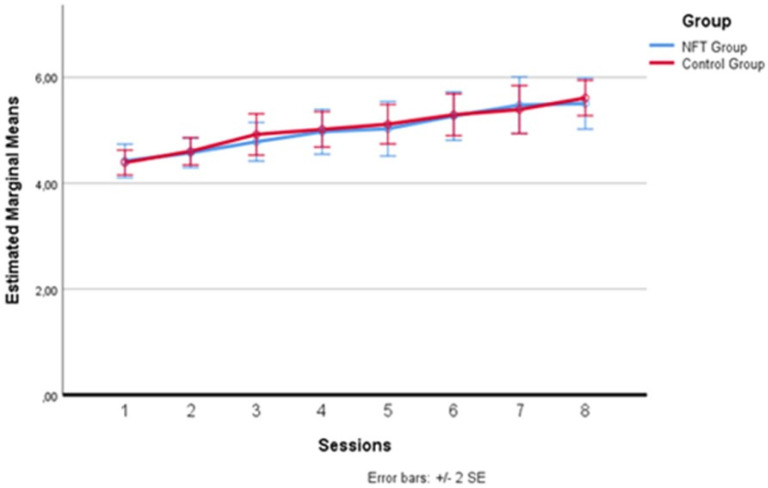
Memory Span performance trends. Progressive trends in Memory Span scores across sessions for both experimental and control groups, indicating a general learning effect without significant differences attributable to neurofeedback training.

Figure 5 below illustrates with greater clarity the variations in the two groups. As can be observed, there was a progressive increase on the mnemonic plane, but the trend is substantially homogeneous between the two groups; hence, no direct effect of the employed neurofeedback training on the working memory of the participants in the experimental group can be associated.

The Corsi Block test, administered in the first and last training session, also did not confirm, through the comparison of values reported in Table 4, the hypothesis of a significant and positive change in mnemonic performance in favor of the experimental group (*p* > 0.05; F = 0.031 partial Eta squared = 0.003).

**Figure 6:**
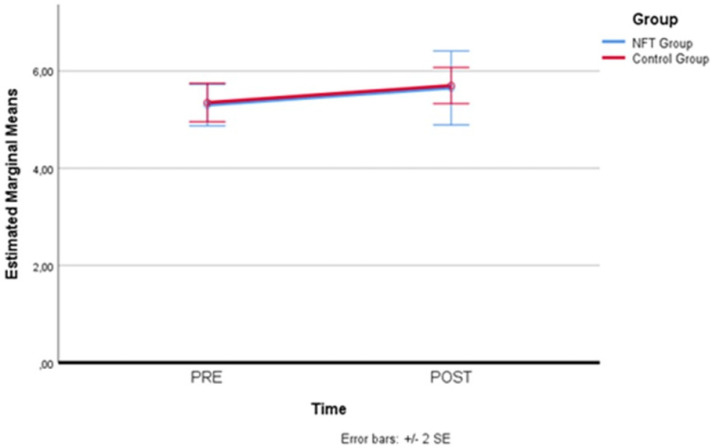
Comparison of Corsi Block test scores. A slight learning effect observed in the Corsi Block test scores from the first to the last session, without significant differences between groups.

Figure 6 clearly shows that there was no difference between the two groups. The slope with an increase in the measure taken in the final session, although less pronounced than what was recorded for the Memory Span evaluation, suggests a slight learning effect of the test. This effect was very pronounced in the trend of the Memory Span scores, also because, in that case, the administrations were carried out on all eight days of the program, which probably facilitated learning of the task, influencing the mnemonic recall values.

Overall, the study observed that the neurofeedback program had a discernible effect on alpha amplitude from the second day. However, unlike findings from other studies in the literature [35,50], it did not confirm the expected concurrent improvement in working memory.

### 3.3. Evaluation of the Intervention Effect on Anxiety Levels

Within the protocol, the measure of general state and trait anxiety in participants was included. The hypothesis was to achieve as a concurrent effect of the neurofeedback intervention on the alpha wave a reduction in general anxiety levels in participants. This effect is well highlighted in numerous works in the literature [51,52,53]. The evaluation was carried out in the first and last training sessions, considering state and trait anxiety. Firstly, a two-way repeated measures ANOVA was run to determine the effect of NFT over time on state anxiety. Analysis of the studentized residuals showed that there was normality, as assessed by the Shapiro–Wilk test of normality, and no outliers, as assessed by no studentized residuals greater than ±3 standard deviations. There was sphericity for the interaction term, as assessed by Mauchly’s test of sphericity (*p* > 0.05). There was a statistically significant interaction between NFT and time on state anxiety, F(1,19) = 61.69, *p* < 0.001, partial η^2^ = 0.765. The contrast analysis, shown in Table 5, reveals a significant interactive effect between the duration of the treatment and the group membership, with effect size measures of 0.342 (partial Eta squared) for the group and 0.822 for the duration of the treatment.

Therefore, simple main effects were run. State anxiety was not statistically significantly different in the control group (M = 29.35, SD = 1.09) compared to the experimental group (M = 29.25, SD = 1.02) at the beginning (pre-) of the NFT, F(1,19) = 0.02, *p* = 0.886, partial η^2^ = 0.001. However, state anxiety resulted statistically significantly different in the experimental group (M = 24.45, SD = 1.02) compared to the controls (M = 28.90, SD = 1.01) at the end of the NFT (post-), F(1,19) = 27.17, *p* = 0.000, partial η^2^ = 0.978, a mean difference of −4.45, 95% CI [−6.24, −2.66]. Therefore, the pre–post comparison between the experimental group and the control group recorded a significant reduction in state anxiety levels in the participants of the experimental group. Table 6 below shows the pre–post state anxiety values recorded in the two groups. Means, standard error, and confidence interval are indicated.

**Figure 7:**
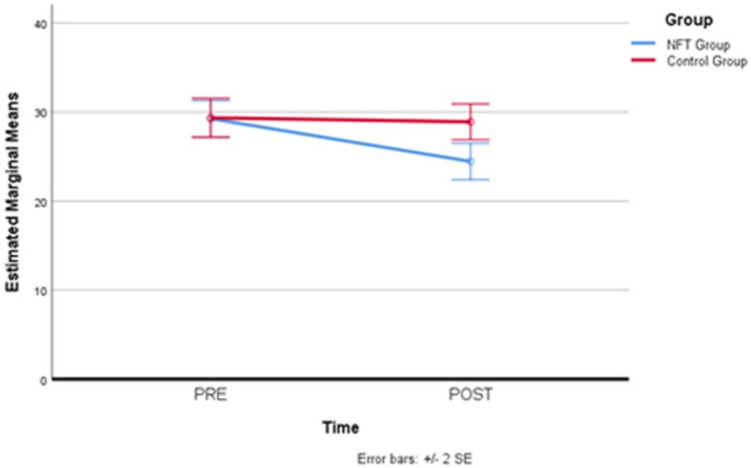
State anxiety level reductions in the experimental group. Significant decrease in state anxiety levels following neurofeedback training, showcasing the intervention’s effectiveness.

Figure 7 below illustrates the positive change in the experimental group, which recorded a significant reduction in state anxiety after eight days.

Also, for trait anxiety, a two-way repeated measures ANOVA was run to determine the effect of NFT over time. Analysis of the studentized residuals showed that there was normality, as assessed by the Shapiro–Wilk test of normality, and no outliers, as assessed by no studentized residuals greater than ±3 standard deviations. There was sphericity for the interaction term, as assessed by Mauchly’s test of sphericity (*p* > 0.05). A statistically significant interaction between NFT and time on trait anxiety resulted: F(1,19) = 9.88, *p* < 0.05, partial η^2^ = 0.342. The contrast analysis, shown in Table 7, reveled a significant interactive effect between the duration of the treatment and the group membership, with effect size measures of 0.599 (partial Eta squared) for the duration of NFT and 0.002 for Group.

**Figure 8:**
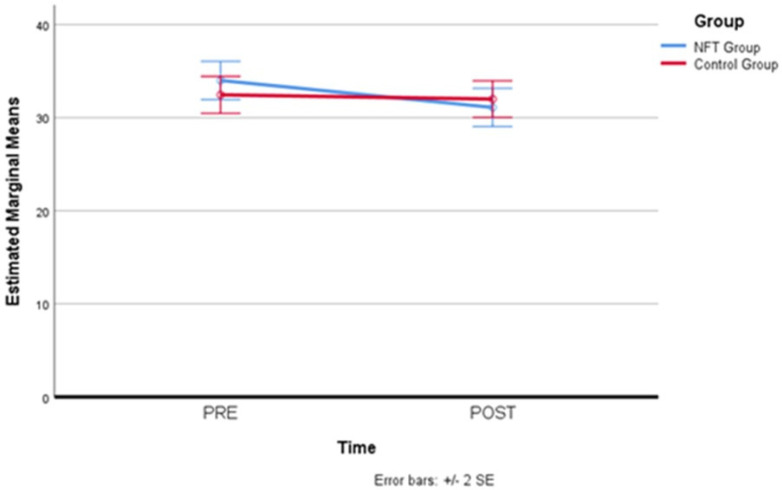
Trait anxiety scores before and after neurofeedback training.

After simple main effects have been run, trait anxiety results were not statistically significantly different in the control group (M = 34.00, SD = 1.03) compared to the experimental group (M = 32.45, SD = 0.99), both at the beginning (pre-) of the NFT, F(1,19) = 0.02, *p* = 0.886, partial η^2^ = 0.001, and at the end of the NFT (post-), F(1,19) = 0.32, *p* = 0.578, partial η^2^ = 0.017, where also no statistical difference in the experimental group (M = 31.10, SD = 1.03) compared to the controls (M = 32.00, SD = 0.98) has been detected. Therefore, for trait anxiety, a reduction in the levels attributable to the neurofeedback treatment did not occur. Table 8 and Figure 8 below report trait anxiety scores for the two groups before and after neurofeedback training.

Overall, the results suggested a possible association between increased alpha amplitude and a significant reduction in state anxiety in the group that underwent the neurofeedback intervention.

## 4. Discussion

The study presented here utilized an economical, commercially available Brain–Computer Interface device (Emotiv Epoc X) as the NFT device to voluntarily control and enhance EEG activity of the relative alpha rhythm (8–12 Hz) and consequently induce an improvement in short-term memory performance and a reduction in anxiety in a single-blind design. In line with previous results [35,50], an enhancing effect of training on relative alpha and short-term memory performance (measured with the Memory Span and Corsi Block tests), as well as a reduction in state and trait anxiety levels (measured with STAI-Y), was expected.

The data indicated an increase in relative alpha in the neurofeedback experimental group from the first to the last recording session, an observation not paralleled in the control group. Additionally, contrast analysis suggested that the increase in alpha amplitude occurred earlier, starting from the second session, for participants who monitored their brain activity in real time compared to those who were exposed to a neutral image. It is noteworthy that after this initial rise, the increase in relative alpha rhythm in the experimental group appeared to stabilize or show slight increments throughout the sessions. The statistically significant results on days 2 and 8, where *p*-values are less than 0.05, suggest that the intervention had a measurable impact on alpha wave amplitude on these specific days. This might indicate that the experimental conditions or the participants’ responses to the neurofeedback training peaked effectively at these times. The lack of statistically significant differences on the other days could suggest several things. The effect of the intervention may have fluctuations due to various factors, such as participant fatigue, adaptation to the neurofeedback, or external variables not controlled within the study. It may also suggest that the changes in alpha amplitude, while possibly present, were not robust enough on these days to be detected as statistically significant against the natural variability within the control group. The significant increases on specific days but not others could be indicative of learning or adaptation effects. It is possible that participants are gradually improving their ability to control or enhance alpha wave activity through neurofeedback, with fluctuations in effectiveness. This could be explored further by analyzing the strategies used by participants on successful days versus non-successful days. Given the variability in results, future studies might look into more extended periods of training, perhaps with additional sessions, to see if a more consistent pattern of alpha wave amplitude increase can be achieved. Examining individual differences in response to neurofeedback could help tailor more effective personalized interventions.

This result could indicate a plateau effect of neurofeedback training, as an initial increase in relative alpha rhythm after the first session was followed by an adaptation that led to stabilizing the new rhythm and not exponentially increasing it. This effect has also been found in previous studies in the literature [35,48,49]. In various studies conducted on healthy participants, it has been reported that the incremental curve of NFT reaches a plateau after 4–6 sessions with a subsequent stagnation (total number of sessions 8–10) [49,54,55,56,57]. It is hypothesized that these plateaus reflect training fatigue or over-learning. Furthermore, learning curve models of a clinical sample might differ from those of healthy subjects. For example, Kübler et al. [58] found that healthy subjects reached a learning plateau after 3 sessions, while in patients with Amyotrophic Lateral Sclerosis, no learning plateau was reached after 12 sessions. In a neurofeedback study on patients with primary insomnia, participants showed fluctuating learning, which, interrupted by stagnation sessions, increased over the sessions [59]. In anxious patients, Hardt and Kamiya [60] hypothesized a fifth-order learning curve, starting with an initial increase, followed by a decline, a second increase, and a final exponential increase for the learning of alpha-neurofeedback.

In general, the results regarding relative alpha demonstrated that training the alpha rhythm with a real-time NFT procedure facilitates the enhancement process of alpha activity. Therefore, the findings of this study are in agreement with some of the most recent studies in the literature [24,35,61], which suggest that neurofeedback training on the alpha rhythm, repeated over several days, produces an increase in relative alpha activity.

The hypothesis, which anticipated an improvement in anxiety levels following NFT on the alpha rhythm compared to the control group, was confirmed. Here again, the results are in line with some of the most recent studies on the subject [50,51,52]. Neurofeedback training on low-frequency brain waves (alpha–theta) allows for a reduction in anxiety and inhibits the activity of specific brain regions, which are determinants in the induction of hyper-arousal [62].

An aspect to highlight in this study is the strategies used for the enhancement of the relative alpha rhythm. In the analysis of the neurofeedback sessions, it was noted that participants commonly used specific mental strategies to enhance their EEG feedback results. These strategies included thinking about emotionally charged subjects, both positive and negative, and visualizing active scenarios such as participating in sports or recalling interactions with loved ones. These observations were derived from qualitative data collected at the end of each neurofeedback session. From the descriptive analysis, it emerged that the most used strategies to obtain feedback activation on the screen were related to thinking about something that evoked emotions, both positive and negative, visualizing oneself in a sporting activity, or thinking about a loved one, a love, or a strong feeling. The analysis of these three strategies on the alpha rhythm, pre–post-training, highlighted two contrasting trends: thinking about positive or negative emotions did not produce a positive increase in the alpha rhythm, while strategies concerning thinking about a situation related to sports activity or a feeling were correlated with an incremental trend of the relative alpha rhythm. This might imply that it is possible to deduce appropriate mental strategies within a session and that consistent visual feedback might not be necessary for subsequent sessions to over-regulate alpha to a certain extent, provided that the appropriate mental strategy is used. This interpretation seems to be in line with studies showing an improvement in alpha from certain types of meditation [63,64]. A replication study with an additional control group, uninformed about which strategies are generally linked to the improvement of the alpha rhythm, could help to further investigate this matter.

Another possible explanation for the improvement in alpha with the aforementioned strategies could be found in the realm of psychophysiological processes. It has been reported in the literature that the alpha rhythm is favored by a relaxed state, and over the course of the study presented here, participants increasingly became familiar with the experimental environment, as well as with the experimenters. It is possible that participants became increasingly relaxed, comfortable, and calm during subsequent neurofeedback sessions, which could have facilitated the improvement of the alpha rhythm in the experimental group. In line with Thibault et al. [65], it might be important to include, in a future study, a control group that works with an inverse neurofeedback program (aimed at reducing the alpha frequency).

An additional result observed in the conducted study is a clear improvement in Memory Span tests over the sessions. However, a significant incremental increase was observed in both groups (experimental and control). Likely, the Memory Span test results were influenced by the learning effect of the tasks. Repeating the Memory Span tests pre and post each session facilitated learning of the task and the adoption of strategies for score improvement. As for the other memory test used, from the analysis of the Corsi Block test scores, there was a linearity between pre- and post-NFT. In this case, the learning effect did not occur, as the scores did not significantly deviate from the initial scores. This result was also obtained because the Corsi Block test, unlike Memory Span, was administered only on two occasions, at the beginning and at the end of the program. These results were observed in both groups and did not confirm our initial hypotheses. Several explanations can be proposed to justify them. Firstly, the rapid improvement in the Memory Span task observed in most participants is most likely due to practice and was stronger than the increase in alpha frequency across the sessions.

The placement of electrodes might also have been responsible for the lack of NFT effect on working memory performance. Feedback signals were acquired from P7, O1, O2, and P8 because the occipital cortex is involved in every visual process, and the parietal sites are connected to attention [66,67]. It is possible that the choice of electrodes could have influenced or compromised the NFT’s effect on memory performance. Many authors have used electrodes in other sites, such as Cz, Pz, Fz, and C3, which differ from the sites used in this study [68,69,70].

The study had some limitations that could have reduced the effectiveness of the planned intervention. Although used in various clinical test batteries and generally considered a useful indicator for cognitive performance, the Memory Span task administered in this study showed rather low intra-individual variation and strong learning effects in the repeated measures chart. It is also possible that the conditioning program was not efficient enough due to the use of a dimensional color code as a feedback signal. Other authors have worked with very specific reward symbols and sounds (for example, acoustic signals, counters, and pleasant sounds [71,72]). Another limitation of the study refers to the software used for NFT, which did not allow for the selection of specific frequency bands. Considering that many studies on working memory improvement have focused on training the individual upper alpha (IUA) [73,74,75], not being able to set the program to such frequency may have reduced the efficacy of the training itself. Another limit of the study could have been using only a relaxing image of the sea for the control group to focus on. It can be observed that for some subjects, this image could evoke relaxing sensations, while for others, it could activate opposite sensations. Thus, for a future study, it might be preferable to include a variety of choices among relaxing images (sea, mountain, etc.) so that everyone can lean toward the one most congenial to their preferences.

One notable limitation of our study pertains to the feedback mechanism used during the neurofeedback sessions. While participants received real-time visual feedback on their alpha wave activity through changes in color intensity on the 3D brain model, our protocol did not include additional reinforcement signals, such as a green checkmark, that could have explicitly indicated successful maintenance of optimal alpha activity levels. This absence might have limited participants’ ability to clearly discern when they had reached or maintained the desired activity level, potentially impacting their motivation and the efficacy of self-regulation strategies. The lack of explicit reinforcement could have implications for the learning and generalization of self-regulation skills beyond the laboratory setting. Participants might have benefited from more structured feedback that not only reflected changes in brain activity but also affirmed correct performance through positive reinforcement cues. Future studies could improve upon this by incorporating a multimodal feedback system that includes both visual and auditory signals to clearly denote when participants achieve or maintain target brain activity levels. This could enhance the training effect by providing immediate and clear reinforcement, thereby potentially increasing the effectiveness of neurofeedback training in real-world applications.

The study used a single-blind design as the experimenters knew which type of group (experimental or control) the participating subjects had been assigned to. In a future study, the hypothesis that even the experimenters are unaware of the group division (double-blind study) should be considered so as not to influence the results, even unconsciously. Moreover, in NFT studies, control groups with Sham feedback are often employed, where subjects in the control group are given false feedback instead of neutral/pleasant stimuli. Planning a Sham control group is considered in the literature as the most appropriate research design for studying an NFT intervention, as it provides a control method for various components that are fundamentally not related to EEG regulation but can nevertheless influence EEG [76,77], including the effects of attention and expectation [78,79]. The non-utilization of a SHAM group as a control is an important limitation, as with the use of a “passive” control (viewing an image) instead of a SHAM group (non-contingent neurofeedback to the target activity), it cannot be discerned whether the observed pre–post changes are indeed due to the modulation of the alpha activity or to the attention and expectations generated by engaging in any activity with a purpose (increasing alpha).

The research project planned and carried out eight daily recording sessions, five consecutive in one week and an additional three consecutive in the following week. This administration mode may have been a limitation, as it likely exposed subjects to fatigue and a significant plateau effect. In future work, it should be considered to schedule interventions with longer breaks between training sessions to foster participants’ motivation and concentration while simultaneously reducing fatigue.

For a study revision, it would also be appropriate to focus on other cognitive functions, such as inhibitory control, attention, attentive focus, and executive functions, in addition to memory. It might also be useful to use other sensors (e.g., frontal and fronto-temporal areas) that concern frontal executive functions, which are widely involved in memory processes [80,81,82].

Further investigation should verify the temporary or lasting and stable nature of the cognitive function outcomes from neurofeedback training. One way to explore this question is to conduct long-term follow-up studies. On this topic, one of the most comprehensive follow-up analyses on neurofeedback was conducted by Tansey et al. [83]. In this study, researchers carried out an NFT protocol of the SMR rhythm of a teenager with ADHD. After 20 sessions of neurofeedback aimed at increasing SMR activity, the boy showed specific improvement in reading and comprehension and a reduction in his hyperactive behavior. An initial follow-up conducted 24 months after treatment revealed that the boy had maintained his behavioral, attentional, and academic progress. After 10 years, a further follow-up showed that the individual continued to demonstrate both academic and personal successes and a normalized EEG profile. Further studies on a larger sample should be planned to verify the long-term efficacy of NFT interventions.

The results of our study revealed that all participants (experimental and control groups) improved in the Memory Span test (performed pre–post for 8 consecutive days) regardless of NFT treatment. This result raises the question of the most suitable intervention method to improve working memory level through NFT.

The findings of this study underscore the significance of tailoring NFT protocols to enhance alpha waves, which have been linked to reduced anxiety and improved cognitive functions, specifically within a university student population. Considering the unique stressors faced by these individuals, including high cognitive demands and the stress associated with academic performance, the application of NFT could represent a targeted strategy to bolster psychological resilience and cognitive efficiency. Our results align with the broader literature on the benefits of NFT in diverse populations but highlight the practical implications of extending these benefits to the educational context.

## 5. Conclusions

This study primarily investigated the impacts of neurofeedback training (NFT) on working memory and anxiety among university students, employing the 14-channel Emotiv Epoc X headset and Brainviz software. Our findings indicate an enhancement in alpha wave amplitude starting from the second day of NFT, suggesting the potential efficacy of this neuro-cognitive technique in improving neural efficiency. Contrary to expectations and the existing literature, no significant improvements were observed in working memory capacities despite the increase in alpha amplitude.

However, the study successfully documented a significant reduction in state anxiety levels among the experimental group. This underscores the potential of NFT not only in cognitive enhancement but also as a formidable tool for anxiety management within an educational context. Such findings advocate for the incorporation of NFT in psychological health strategies, particularly in stress-prone environments like universities.

Despite these promising outcomes, the absence of expected improvements in working memory highlights the complexity of cognitive processes and suggests that alpha wave modulation might not directly correlate with cognitive enhancements as previously perceived. This discrepancy also indicates the necessity for further research to explore the intricate dynamics between different neural activities and cognitive functions.

The application of NFT showed that simpler, cost-effective neurofeedback devices could be practically integrated into regular educational settings, offering a non-invasive method to aid mental health and cognitive performance. Future studies should consider diverse neurofeedback protocols and perhaps integrate multimodal approaches to fully understand and harness the benefits of neurofeedback.

Our findings indicated a significant reduction in state anxiety levels following the combination of neurofeedback training and relaxation techniques. While this reduction is beneficial for managing high anxiety levels, it is important to consider the potential impact of excessively low anxiety on cognitive performance. Cognitive tasks typically require a certain level of cognitive arousal to maintain optimal performance. According to the Yerkes–Dodson law, there is an optimal level of arousal for peak performance, and both insufficient and excessive arousal can impair cognitive functioning. In this study, the particularly low levels of state anxiety achieved may have reduced cognitive arousal to a point where it negatively affected working memory performance. This suggests that while reducing high anxiety to moderate levels can enhance cognitive functions, further reduction to very low levels may not be advantageous and could even hinder performance. Future research should explore the balance between anxiety reduction and cognitive arousal to identify the optimal level for enhancing cognitive performance in educational and therapeutic settings.

While these results suggest some potential of the technique in enhancing neural efficiency, the variability across different days highlights the need for further investigation to fully ascertain its effectiveness. The study confirms the beneficial impact of NFT on reducing state anxiety among university students, underscoring its value in psychological and cognitive performance enhancement. Despite the lack of observed improvements in working memory, these results highlight the need for continued exploration of NFT applications across different populations and settings, emphasizing its potential utility in educational and therapeutic contexts.

## Figures and Tables

**Table 1:** Group and Time alpha wave amplitudes.

Group	Time	Mean	Std. Error	Confidence Interval 95%	
				Lower Limit	Upper Limit
Experimental Group	1	0.659	0.037	0.581	0.736
	2	0.820	0.050	0.716	0.924
	3	0.751	0.044	0.658	0.844
	4	0.803	0.049	0.700	0.905
	5	0.769	0.046	0.674	0.865
	6	0.790	0.026	0.736	0.844
	7	0.791	0.032	0.725	0.857
	8	0.830	0.035	0.756	0.904
Control Group	1	0.638	0.041	0.553	0.724
	2	0.658	0.055	0.554	0.782
	3	0.663	0.051	0.556	0.770
	4	0.672	0.051	0.566	0.778
	5	0.671	0.038	0.591	0.751
	6	0.656	0.062	0.526	0.786
	7	0.652	0.061	0.525	0.779
	8	0.637	0.049	0.533	0.740

**Table 2:** Group alpha wave pairwise comparisons across the eight days.

Day	Mean Difference	Standard Error	Lower Limit	Upper Limit	F	*p*-Value	Partial Eta Squared
1	0.021	0.052	−0.089	0.130	0.154	0.699	0.008
2	0.162 *	0.049	0.018	0.221	6.054	0.024	0.242
3	0.751	0.074	−0.067	0.244	1.431	0.246	0.070
4	0.130	0.072	−0.021	0.282	3.239	0.088	0.146
5	0.098	0.066	−0.041	0.066	2.183	0.156	0.103
6	0.134	0.066	−0.004	0.272	4.111	0.057	0.178
7	0.139	0.073	−0.013	0.292	3.652	0.071	0.161
8	0.193 *	0.059	0.069	0.318	10.564	0.004	0.357

*Note.* * = *p* < 0.05.

**Table 3:** Group and Time Memory Span performances.

Group	Time	Mean	Std. Error	Confidence Interval 95%	
				Lower Limit	Upper Limit
Experimental Group	1	4.419	0.158	4.088	4.751
	2	4.572	0.140	4.279	4.864
	3	4.781	0.182	4.400	5.163
	4	4.970	0.212	4.526	5.414
	5	5.027	0.257	4.489	5.565
	6	5.267	0.228	4.789	5.744
	7	5.475	0.267	4.916	6.034
	8	5.504	0.242	4.998	6.010
Control Group	1	4.389	00.117	4.143	4.634
	2	4.601	0.129	4.331	4.871
	3	4.920	0.196	4.511	5.329
	4	5.015	0.167	4.666	5.364
	5	5.113	0.186	4.725	5.502
	6	5.292	0.198	4.879	5.705
	7	5.390	0.227	4.916	5.864
	8	5.609	0.167	5.259	5.959

**Table 4:** Corsi Block test performance before and after intervention.

Group	Time	Mean	Std. Error	Confidence Interval 95%	
				Lower Limit	Upper Limit
Experimental Group	Pre	5.300	0.213	4.817	5.783
	Post	5.650	0.380	4.789	6.511
Control Group	Pre	5.350	0.198	4.902	5.798
	Post	5.700	0.186	5.280	6.120

**Table 5:** Interactive effects of treatment duration and Group on state anxiety values.

Origin	Type III Sum of Squares	df	Quadratic Mean	F	Sign.	Partial Eta Squared
Group	103.513	1	103.513	9.871	0.005	0.342
Error (Group)	199.237	19	10.486			
Time	137.813	1	137.813	87.463	0.000	0.822
Error (Time)	29.938	19	1.576			
Group × Time	94.613	1	94.613	61.695	0.000	0.765
Error (Group × Time)	29.137	19	1.534			

**Table 6:** State anxiety values pre–post-intervention.

Group	Time	Mean	Std. Error	Confidence Interval 95%	
				Lower Limit	Upper Limit
Experimental Group	Pre	29.25	1.018	27.119	31.381
	Post	24.45	1.025	22.305	26.595
Control Group	Pre	29.35	1.094	27.061	31.639
	Post	28.90	1.005	26.797	31.003

**Table 7:** Trait anxiety values pre–post-intervention.

Origin	Type III Sum of Squares	df	Quadratic Mean	F	Sign.	Partial Eta Squared
Group	2.112	1	2.112	0.046	0.832	0.002
Error (Group)	871.637	19	45.876			
Time	56.113	1	56.113	28.326	0.000	0.599
Error (Time)	37.637	19	1.981			
Group × Time	30.013	1	30.013	9.876	0.005	0.342
Error (Group × Time)	57.737	19	3.039			

**Table 8:** Trait anxiety values before and after NFT.

Group	Time	Mean	Std. Error	Confidence Interval 95%	
				Lower Limit	Upper Limit
Experimental Group	Pre	34.00	1.026	31.853	36.147
	Post	31.10	1.026	28.953	33.247
Control Group	Pre	32.45	0.993	30.371	34.529
	Post	32.00	0.979	29.952	34.048

## Data Availability

The raw data supporting the conclusions of this article will be made available by the corresponding author on request. The data are not publicly available due to specific ethical and privacy considerations.
